# TNPO3 protects HIV-1 replication from CPSF6-mediated capsid stabilization in the host cell cytoplasm

**DOI:** 10.1186/1742-4690-10-20

**Published:** 2013-02-15

**Authors:** Alberto De Iaco, Federico Santoni, Anne Vannier, Michel Guipponi, Stylianos Antonarakis, Jeremy Luban

**Affiliations:** 1 Department of Microbiology and Molecular Medicine, University of Geneva, 1205, Geneva, Switzerland; 2 Department of Genetic Medicine and Development, University of Geneva, 1205, Geneva, Switzerland; 3 Program in Molecular Medicine, University of Massachusetts Medical School, 373 Plantation Street, Biotech II, Suite 319, 01605, Worcester, MA, USA

**Keywords:** HIV-1, TNPO3, CPSF6, Capsid, Nuclear transport

## Abstract

**Background:**

Despite intensive investigation the mechanism by which HIV-1 reaches the host cell nucleus is unknown. TNPO3, a karyopherin mediating nuclear entry of SR-proteins, was shown to be required for HIV-1 infectivity. Some investigators have reported that TNPO3 promotes HIV-1 nuclear import, as would be expected for a karyopherin. Yet, an equal number of investigators have failed to obtain evidence that supports this model. Here, a series of experiments were performed to better elucidate the mechanism by which TNPO3 promotes HIV-1 infectivity.

**Results:**

To examine the role of TNPO3 in HIV-1 replication, the 2-LTR circles that are commonly used as a marker for HIV-1 nuclear entry were cloned after infection of TNPO3 knockdown cells. Potential explanation for the discrepancy in the literature concerning the effect of TNPO3 was provided by sequencing hundreds of these clones: a significant fraction resulted from autointegration into sites near the LTRs and therefore were not *bona fide* 2-LTR circles. In response to this finding, new techniques were developed to monitor HIV-1 cDNA, including qPCR reactions that distinguish 2-LTR circles from autointegrants, as well as massive parallel sequencing of HIV-1 cDNA. With these assays, TNPO3 knockdown was found to reduce the levels of 2-LTR circles. This finding was puzzling, though, since previous work has shown that the HIV-1 determinant for TNPO3-dependence is capsid (CA), an HIV-1 protein that forms a mega-dalton protein lattice in the cytoplasm. TNPO3 imports cellular splicing factors via their SR-domain. Attention was therefore directed towards CPSF6, an SR-protein that binds HIV-1 CA and inhibits HIV-1 nuclear import when the C-terminal SR-domain is deleted. The effect of 27 HIV-1 capsid mutants on sensitivity to TNPO3 knockdown was then found to correlate strongly with sensitivity to inhibition by a C-terminal deletion mutant of CPSF6 (R^2^ = 0.883, p < 0.0001). TNPO3 knockdown was then shown to cause CPSF6 to accumulate in the cytoplasm. Mislocalization of CPSF6 to the cytoplasm, whether by TNPO3 knockdown, deletion of the CPSF6 nuclear localization signal, or by fusion of CPSF6 to a nuclear export signal, resulted in inhibition of HIV-1 replication. Additionally, targeting CPSF6 to the nucleus by fusion to a heterologous nuclear localization signal rescued HIV-1 from the inhibitory effects of TNPO3 knockdown. Finally, mislocalization of CPSF6 to the cytoplasm was associated with abnormal stabilization of the HIV-1 CA core.

**Conclusion:**

TNPO3 promotes HIV-1 infectivity indirectly, by shifting the CA-binding protein CPSF6 to the nucleus, thus preventing the excessive HIV-1 CA stability that would otherwise result from cytoplasmic accumulation of CPSF6.

## Background

Early events in the human immunodeficiency virus type 1 (HIV-1) replication cycle commence when HIV-1 binds and fuses to a susceptible host cell. With fusion, the conical HIV-1 virion core is released into the cytosol. The virion core measures 119 × 60 nm on average [1] and bears a proteinaceous coat of more than 1,000 capsid monomers [2]. The viral genomic RNA within the coat serves as template for the viral reverse transcriptase (RT), in a process that links disassembly of the virion core to viral cDNA synthesis [3]. The resulting pre-integration complex (PIC) then gains access to the nucleus, presumably through the nuclear pore complex, where the viral integrase (IN) ligates the viral cDNA to host cell chromosomal DNA.

Exactly how the viral genome reaches the nucleus of the host cell is not clear. Correlations between biochemistry and function are rendered difficult by the fact that only a minority of retrovirion particles within any given preparation succeed in transducing susceptible target cells [4]. Genetic approaches have been fruitful in that several cellular factors have been identified that influence early HIV-1 replication steps occurring after reverse transcription [5-10]. One such factor, Transportin-3 (TNPO3), is a karyopherin that imports SR-rich splicing factors into the nucleus [11].

TNPO3 clearly plays an important, though controversial role in HIV-1 replication [5,6,8,9,12-16]. Some studies suggest that TNPO3 promotes nuclear import of the PIC [6,9,14,17]. Others indicate that it has a role in a step after the viral PIC reaches the nucleus [8,12,13,15,16]. TNPO3 has been reported to interact with both IN and CA [6,13,16,18], but the relevance for HIV-1 replication of the interaction with IN was not confirmed [15,19]. Evidence supporting the functional significance of interaction with CA is stronger than that for interaction with IN. MLV does not require TNPO3 for transduction; chimeras in which HIV-1 CA and IN are swapped with the MLV counterparts reveal a central role for CA in TNPO3 function and fail to demonstrate a role for IN [19]. Additionally, nearly 30 HIV-1 CA mutants have been identified that alter HIV-1 dependence on TNPO3 [7,8,13,19]. How TNPO3 would promote HIV-1 infectivity via effects on CA is not obvious. Shah et al. suggests that TNPO3 acts directly on the process of CA core uncoating [20].

Cleavage and polyadenylation specific factor 6 (CPSF6) is a 68-kD subunit of the mammalian cleavage factor I (CF Im), a component of the mRNA cleavage/polyadenylation machinery [21]. CPSF6 possesses an N-terminal RNA recognition motif (RRM), a central proline-rich domain, and a C-terminal domain enriched in arginine/serine, arginine/glutamate and arginine/aspartate repeats, similar to the RS-domain of SR splicing factors [22]. A CPSF6 C-terminal deletion mutant lacking the RS-like domain (CPSF6-358) was isolated in an expression screen for cDNAs that inhibit HIV-1 replication [7]. CPSF6-358 binds HIV-1 CA and viral restriction activity depends on this interaction [7,23]. Closer examination of CPSF6 revealed a number of functional links with TNPO3. The CPSF6 RS-like domain is required for localization to the nuclear compartment [7,24]. Interestingly, TNPO3 imports cargo proteins by interacting with the RS-domain of SR-proteins [25]. CPSF6-358 binds specifically an HIV-1 CA pocket where amino acids important in HIV-1 dependency to TNPO3 are located [7,8,23]. HIV-1, HIV-2 and SIV macaque (SIVmac), but not MLV and FIV, are inhibited by CPSF6-358 expression and TNPO3 depletion [6,7,19].

Here we set out to clarify the mechanism by which TNPO3 promotes HIV-1 infectivity. We began by pinpointing the step in the HIV-1 replication cycle that is blocked by TNPO3 knockdown and then sought evidence for functional links between HIV-1, TNPO3, and CPSF6.

## Results

### TNPO3 depletion blocks HIV-1 replication in a step before the virus enters the nucleus

Several groups reported experiments designed to determine at which step HIV-1 replication is blocked when TNPO3 is depleted [5,6,8,9,12-17]. All observed a block after completion of reverse transcription but results differed with respect to the effects of TNPO3 knockdown on the formation of 2-LTR circles, a marker for nuclear import of the retrovirus replication machinery [26]. Some studies showed that TNPO3 acts to promote integration, without effects on the levels of 2-LTR circles [8,12,13,15,16]. Other studies reported that the levels of 2-LTR circles were decreased, indicating that TNPO3 is required before nuclear import [6,9,14,17].

**Figure 1:**
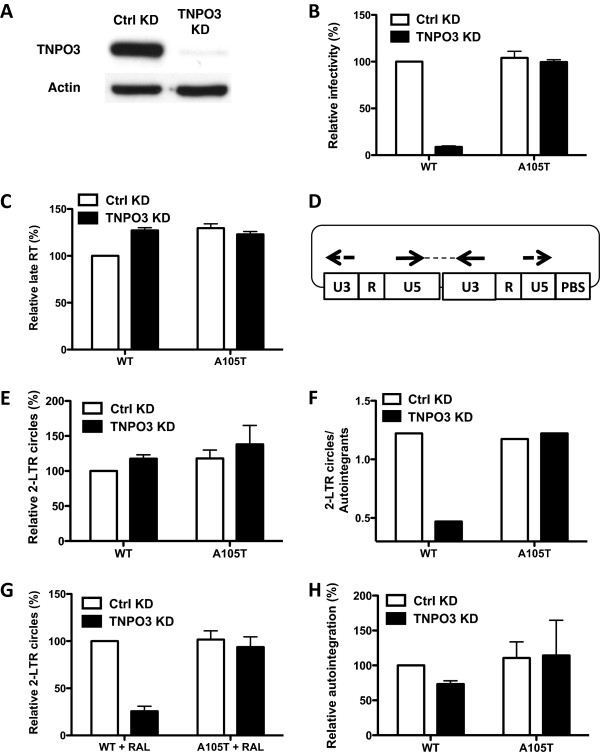
**TNPO3 depletion decreases the level of HIV-1 2-LTR circles and has no effect on autointegration.** (**A**) TNPO3 protein in TZM-bl cells transduced with lentiviral vectors expressing shRNAs targeting TNPO3 or control (Ctrl). Cell lysate was probed in western blots with anti-TNPO3 antibody (upper panel) or anti-β-actin antibody (lower panel). (**B**) TZM-bl control (Ctrl) cells and TNPO3 KD cells were challenged with HIV-1-GFP reporter vectors bearing either WT or A105T CA mutation. 72 hrs after transduction, the percent GFP^+^ cells was determined by flow cytometry as an indication of infectivity. (**C**) Quantification of late RT products 24 hrs post-infection with WT or A105T CA mutant viruses, on control (Ctrl) or TNPO3 KD cells. (**D**) Schematic representation of qPCR for 2-LTR circles using primers flanking the circle junction. Arrows represent primers. Dashed line represents the PCR product. (**E**) Quantification of 2-LTR circles (as indicated in B) 24 hrs post-infection with WT or A105T CA mutant viruses, on control (Ctrl) or TNPO3 KD cells. The PCR products were detected using Sybr green. (**F**) The PCR products from (**E**) were cloned and sequenced. The ratio between 2-LTR circles and autointegration events is plotted. (**G**) The analysis from (**E**) was repeated in the presence of raltegravir (RAL) 10 μM. (**H**) Level of autointegration events by qPCR when WT or A105T CA mutant viruses infect control (Ctrl) or TNPO3 KD cells.

To clarify which step in the HIV-1 replication cycle is promoted by TNPO3 we conducted a more extensive analysis than previously reported. TNPO3 was knocked down in HeLa TZM-bl cells (Figure 1A). These cells were challenged with *env*-, VSV G-pseudotyped, HIV-1_NL4-3_ vectors carrying either WT CA or the A105T CA mutation. Expression of the GFP reporter gene was assessed by flow cytometry 72 hrs after infection, as an indicator of viral infectivity (Figure 1B). As previously shown, depletion of TNPO3 from the host cell resulted in inhibition of WT virus replication, but no effect on the replication of the A105T CA mutant was observed (Figure 1B) [8]. Also as expected [8], there was no effect of the TNPO3 KD on reverse transcription by the WT HIV-1 (Figure 1C).

Then, the levels of 2-LTR circles were assessed 24 hrs post-infection with the VSV G-pseudotyped HIV-1_NL4-3_. The PCR primers were commonly used sequences that flank the junction of the ligated, viral cDNA termini, but do not overlap with the junction (Figure 1D) [8,26]. Despite an 11-fold decrease in the efficiency of transduction of TNPO3 KD cells by WT virus, as compared with the TNPO3-independent A105T CA mutant virus, the replication of which is TNPO3-independent (Figure 1B), no significant reduction in the amount of 2-LTR circles was observed (Figure 1E).

PCR products from the reactions designed to detect 2-LTR circles were ligated into a plasmid and at least 85 clones from each condition were sequenced and evaluated, as previously described [27,28]. 2-LTR circles with consensus sequence were observed, as well as point mutations, insertion of PBS or PPT sequences at the junctions, and deletions at the termini of the viral cDNA (Additional file 1: Table S1). Autointegration resulting from opposite-strand joining was also detected [29,30]; these circular viral cDNAs contain 2-LTRs but are distinguished from 2-LTR circles that result from end-joining by the lack of the terminal dinucleotides that are removed by IN when the viral cDNA is inserted into various regions of the viral genome.

Since the PCR reaction that most labs use to amplify 2-LTR circles also amplified significant quantities of autointegrants, the effect of TNPO3 KD on the signal in this reaction necessitated adjustment for the quantity of autointegrants. The relative amount of 2-LTR circles produced by WT virus, divided by the amount of the opposite-strand autointegrants, was decreased roughly 10-fold by TNPO3 KD (Figure 1F). No significant change in this adjusted ratio was observed with the TNPO3-independent A105T virus. To determine if this change in ratio was due to an absolute decrease in 2-LTR circles or to an increase in autointegrants, TNPO3 KD and control cells were transduced in the presence of raltegravir, a drug that blocks integration. Under this condition the level of 2-LTR circles was reduced 4-fold in the absence of TNPO3 (Figure 1G). Autointegration was assessed independently using a PCR method developed by Yan et al. [31]; when WT or A105T capsid mutant viruses were used to transduce either control or TNPO3 KD cells, the levels of autointegrants were very similar (Figure 1H). Autointegration was not detected when WT virus was used to infect raltegravir-treated cells (data not shown). These experiments indicate that, in the absence of the confounding variable of autointegration, TNPO3 KD was associated with an absolute decrease in the level of 2-LTR circles.

**Figure 2:**
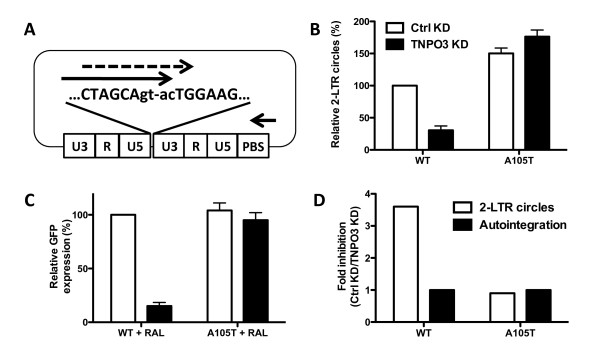
**PCR with oligonucleotides that overlap the circle junction demonstrate decreased levels of HIV-1 2-LTR circles when TNPO3 is depleted.** (**A**) Schematic representation of 2-LTR circle qPCR in which the forward primer overlaps with the circle junction. In particular the forward primers anneal with the 3′LTR and either 2 (solid arrow, Junct2 fwd) or 4 (dashed arrow, Junct4 fwd) nucleotides of the 5′LTR. (**B**) Quantification of 2-LTR circle PCR products with forward primer overlapping 2 nucleotides of the 5′LTR (as indicated in G, Junct2 fwd), 24 hours after infection of WT and A105T CA mutant viruses on control (Ctrl) or TNPO3 KD TZM-bl cells. The PCR products were detected using Sybr green. (**C**) Env- HIV-1 viruses carrying either WT or A105T mutated CA and encoding GFP-reporter (HIV-1_NL4-3_GFP) were used to challenge control (Ctrl) and TNPO3 KD cells treated with 10 μM raltegravir. At 48 hrs the percent GFP^+^ cells was determined by flow cytometry as an indication of GFP expression from unintegrated viruses. (**D**) 2-LTR circles and autointegration events quantified by high-throughput sequencing of low-molecular weight DNA extracted from control (Ctrl) or TNPO3 KD cells, 24 hrs after transduction with WT or A105T CA mutant viruses. Ratios between the levels in control (Ctrl) KD versus TNPO3 KD are plotted. Data represent one of at least three independent experiments. Error bars represent ± SEM (n = 3).

Given that the standard 2-LTR circle primers (Figure 1C) [8,26] detect autointegrants, and that the results of this assay were confounded significantly by this variable, assays were developed that are specific for 2-LTR circles. When the forward PCR primer extended across the junction between the 2-LTR circles such that hybridization would be disrupted by the terminal nucleotide deletion characteristic of autointegration (Figure 2A), 2-LTR circle formation was decreased by TNPO3 KD; Figure 2B shows results using a primer that crossed 2 nucleotides over the circle junction (junct2 fwd) and Additional file 1: Figure S1A shows results with a primer that crosses the junction by 4 nucleotides (junct4 fwd). With these primers, in the presence of raltegravir, the amount of 2-LTR circles increased 10-fold (Additional file 1: Figure S1B), even in control KD cells; with the conventional primers [8,26] there was no significant change with raltegravir, apparently because any increase in 2-LTR circles was canceled by decrease in autointegrants. Similar reduction in 2-LTR circles with TNPO3 KD was observed when PCR products from the conventional 2-LTR primers [8,26] were detected with a TaqMan probe that hybridized across the 2-LTR circle junction (Additional file 1: Figure S1B). In contrast, TNPO3 KD had no effect on 2-LTRs when a TaqMan probe external to the circle junction was used (Additional file 1: Figure S1C) [26].

To confirm a block prior to the formation of 2-LTR circles, control and TNPO3 KD cells were treated with raltegravir, in order to block viral integration. These cells were then challenged with HIV-1_NL4-3_-based vectors encoding the green fluorescence protein (GFP) reporter gene and carrying WT or A105T capsid mutation (Figure 2C). Expression of the reporter gene from the unintegrated viral DNA was detectable 48 hrs after infection, though the mean fluorescence intensity was much weaker than the signal in the absence of raltegravir, and it disappeared if cells were cultured for more than 7 days (data not shown). This signal required de novo cDNA synthesis in the target cells since GFP was not detected if reverse transcription was blocked by AZT treatment of the target cells, or by mutation of the catalytic site of RT (D185K/D186L) in the challenging vector (data not shown). GFP expression from WT virus was strongly reduced when TNPO3 was depleted, while the A105T CA mutant virus was independent. These results clearly show that TNPO3 KD blocks HIV-1 in a step before 2-LTR circles are formed.

Finally, the effect of TNPO3 depletion on 2-LTR circles and autointegration was assessed by high-throughput sequencing (Figure 2D). The complete high throughput sequencing dataset has been submitted to the Sequence Read Archive (SRA), http://www.ncbi.nlm.nih.gov/sra, under accession number SRA056122. TNPO3 KD and control cells were infected with WT and A105T viruses for 24 hours. Low-molecular weight DNA was extracted from the infected cells, primed with random hexamers, and amplified by PCR. The reads were first aligned to human DNA, and the unmapped reads were extracted and remapped to the HIV-1 genome. Then, only reads encompassing a junction were considered (Additional file 1: Table S2). That is, reads having two mapping points on the HIV-1 genome, such as LTR/LTR or LTR/other location, were considered. Finally, junctions were classified according to position and whether the LTR terminal CA was maintained (circles) or cleaved (autointegrants). High-throughput sequencing of the PCR products detected ~10^5^ reads with definitive LTR sequences for each condition, of which, 91% were autointegrants. (Figure 2D). The level of autointegration was similar in WT and A105T CA mutant virus infecting either control or TNPO3 KD cells. 2-LTR circles were significantly reduced when WT virus infected TNPO3 KD cells, while A105T CA mutant virus was totally independent. These results show unequivocally that TNPO3 depletion inhibits HIV-1 replication in a step prior to 2-LTR circle formation.

### Tight correlation between the effects of TNPO3 depletion and CPSF6-358 on transduction by a panel of 27 HIV-1 CA mutants

**Figure 3:**
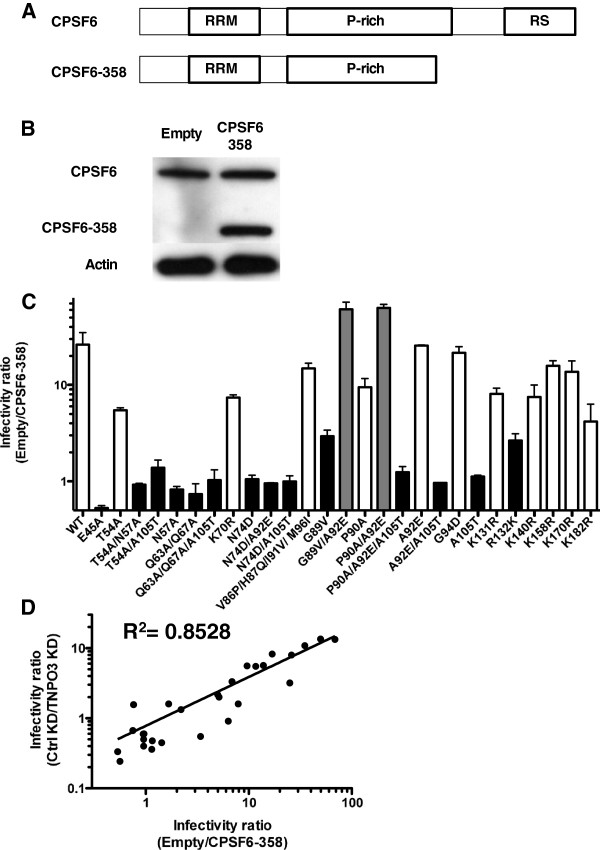
**The effect of CPSF6-358 on the infectivity of HIV-1 CA mutants correlates with the effect of TNPO3 KD.** (**A**) Schematic representation of the protein domains of WT CPSF6 and the truncated mutant CPSF6-358. RNA recognition motif (RRM), proline-rich domain (P-rich), arginine/serine rich domain (RS). (**B**) Expression levels of CPSF6 in TZM-bl cells transduced with empty or CPSF6-358 vectors. Cell lysates were probed in western blots with anti-CPSF6 antibody (upper panel) and anti-β-actin antibody (lower panel). The upper panel shows the endogenous CPSF6 and the truncated form. (**C**) TZM-bl cells transduced with an empty vector or with a vector encoding CPSF6-358 were challenged with a panel of 27 HIV-1-GFP reporter vectors bearing either WT CA or the indicated CA mutants. At 72 hrs the percent GFP^+^ cells was determined by flow cytometry as an indication of infectivity. The ratio of HIV-1 infectivity in CPSF6-358 expressing cells and empty vector cells is shown. White bars show CA mutants inhibited to a similar extent as the WT virus by CPSF6-358, black bars shows CA mutants insensitive or slightly sensitive to CPSF6-358, and gray bars show CA mutants hypersensitive to the presence of CPSF6-358 in the cell. (**D**) Correlation between the infectivity ratios of the 27 CA mutants when infecting Ctrl KD vs TNPO3 KD [8] and Empty vector vs CPSF6-358 (R^2^ = 0.8528).

TNPO3 depletion inhibits HIV-1 transduction in a CA-specific manner [8,19]. Assessment of a panel of 27 HIV-1 CA mutants found that 2 CA mutants were hypersensitive to TNPO3 KD, and 14 CA mutants were TNPO3-independent [8]. Among the 14 TNPO3-independent mutants, E45A, Q63A/Q67A, and N74D were also reported to be resistant to CPSF6-358, a C-terminal truncation mutant of the mRNA processing factor CPSF6 (Figure 3A) that was isolated in an expression screen for cDNAs that inhibit HIV-1 transduction [7]. Using the same methods described above for assessment of the effect of TNPO3 KD, including cloning and sequencing of PCR products, PCR with primers that distinguish 2-LTR circles from autointegrants, and deep sequencing of viral cDNA, CPSF6-358 was demonstrated to inhibit HIV-1 replication at a stage in the HIV-1 replication cycle before 2-LTR circles are formed (Additional file 1: Figure S1D-1G). These findings agree with the replication block reported in ref [7].

To determine if HIV-1 determinants for sensitivity to CPSF6-358 track with those for TNPO3-dependence, the infectivity of each of the 27 previously characterized HIV-1 CA mutants [8] was assessed on cells expressing CPSF6-358 (Figure 3C). HeLa TZM-bl cells were challenged with a bicistronic, lentiviral vector encoding CPSF6-358 and puromycin acetyl transferase (PAT), or with the parental, control vector encoding only PAT. Pools of the two transduced cell populations were selected with puromycin (Figure 3B) and challenged with VSV G-pseudotyped, 3-part, lentiviral vectors, in which the GFP-encoding reporter genome and the viral structural proteins are provided by separate plasmids.

Transduction efficiency by vector bearing wild-type CA was reduced 26-fold by CPSF6-358 (Figure 3C). 11 CA mutants were inhibited to similar extent as the wild type (represented by white bars in Figure 3C). Vectors bearing the CA mutants G89V/A92E or P90A/A92E displayed greater sensitivity to CPSF6-358 than the wild type (gray bars, up to 60-fold lower in the presence of CPSF6-358, as compared to control cells). Fourteen CA mutants were insensitive to the inhibitory effect of CPSF6-358 (black bars).

As compared with HIV-1 CA determinants for sensitivity to TNPO3 depletion [8], sensitivity to inhibition by CPSF6-358 tracked closely (R^2^ = 0.883, p < 0.0001; Figure 3D). In contrast, no significant correlation for the effect of CPSF6-358 was found when the 27 CA mutants were tested on the CA-dependent HIV-1 restriction factors human TRIM5Cyp (hTRIMCyp) or rhesus monkey TRIM5α (rhTRIM5α) (Additional file 1: Figure S2) [32,33]. Such tight correlation in the behavior of the CA mutants is consistent with a common mechanism of action for HIV-1 inhibition by TNPO3 KD and CPSF6-358.

### Cytoplasmic localization of CPSF6-358 is required to inhibit HIV-1

**Figure 4:**
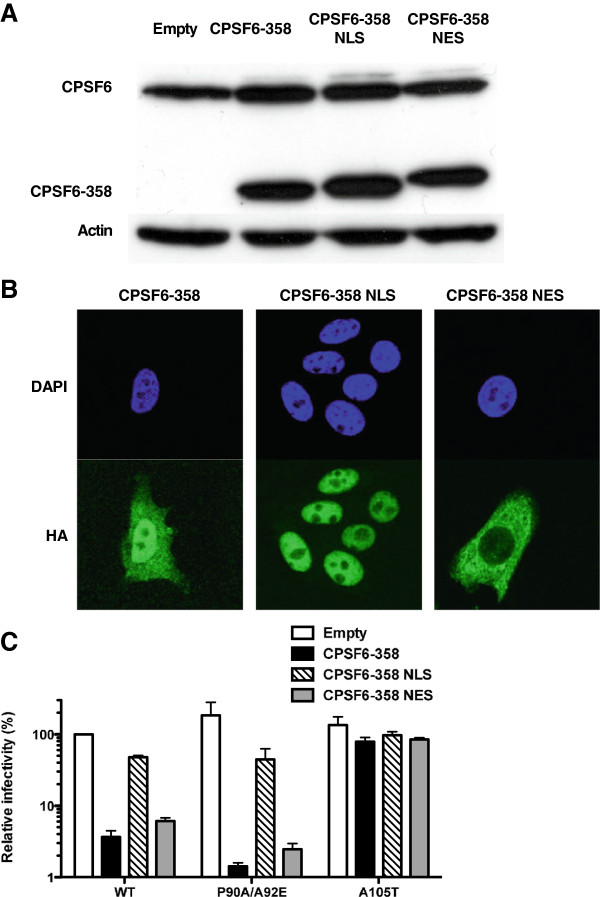
**CPSF6-358 inhibits HIV-1 replication when localized to the cytoplasm.** (**A**) CPSF6 protein in TZM-bl cells transduced with empty, CPSF6-358, CPSF6-358 NLS and CPSF6-358 NES vectors. Cell lysates were probed in western blots with anti-CPSF6 antibody (upper panel) and anti-β-actin antibody (lower panel). The upper panel shows the endogenous CPSF6 and the truncated form. (**B**) Indirect immunofluorescence showing localization of the different forms of CPSF6-358. The TZM-bl cells, transduced as in (**A**), were stained with an anti-HA antibody (green) for the detection of the CPSF6-358 proteins. DAPI was used to mark the nuclear compartment (blue). (**C**) TZM-bl stably expressing the different forms of CPSF6-358 were challenged with WT or CA mutant HIV-1_NL4-3_GFP reporter viruses. After 72 hrs, GFP reporter expression was assessed by flow cytometry. Data represent one of at least three independent experiments. Error bars represent ± SEM (n = 3).

CPSF6 possesses a C-terminal arginine/serine (RS) domain (Figure 3A) that acts as a nuclear localization signal (NLS) for nuclear splicing factors [11]. The CPSF6-358 truncation mutant lacks the RS domain and, unlike full-length CPSF6, is readily detectable in the cytosol [7]. To determine if CA-specific inhibition of HIV-1 correlates with mis-localization of CPSF6-358 to the cytoplasm, HeLa TZM-bl cells were transduced with bi-cistronic lentiviral vectors encoding CPSF6-358, or CPSF6-358 fused to either the SV40 T-Ag NLS or the cyclic AMP-dependent protein kinase inhibitor (PKI) nuclear export signal (NES). The C-terminus of each CPSF6-358 protein was fused to the influenza hemaglutinin (HA) epitope. Expression of the three CPSF6-358 fusion proteins was assessed by western blot with anti-HA antibody (Figure 4A). The HA epitope was also used to assess subcellular localization of the proteins by indirect immunofluorescence; CPSF6-358 was distributed throughout the cell, CPSF6-358-NLS was only detected in the nuclear compartment, and CPSF6-358-NES was primarily detected in the cytosol (Figure 4B).

The three pools of cells with CPSF6-358 targeted to different cellular compartments were then challenged with HIV-1 vectors bearing WT CA, A105T CA, or P90A/A92E CA (Figure 4C). Virus bearing the A105T CA mutant was resistant to the inhibitory effects of CPSF6-358, whether the HIV-1 inhibitor was localized to the nucleus or to the cytoplasm. Amino acid A105 is in fact localized to the pocket where CPSF6 binds to HIV-1 CA and makes direct contact with the SR-protein [23]. Alteration of the A105 side chain in the A105T mutant would be expected to disrupt the interaction between CA and CPSF6 and the consequent viral restriction would be abolished. Infection with WT or P90A/A92E CA mutant viruses was inhibited when CPSF6-358 was detected in the cytosol. When CPSF6-358 was targeted to the nucleus by fusion to the SV40 NLS, HIV-1 transduction was no longer inhibited, indicating that CPSF6-358 needs to be in the cytoplasm to inhibit HIV-1 infection.

### HIV-1 is inhibited when full-length CPSF6 is targeted to the cytoplasm

**Figure 5:**
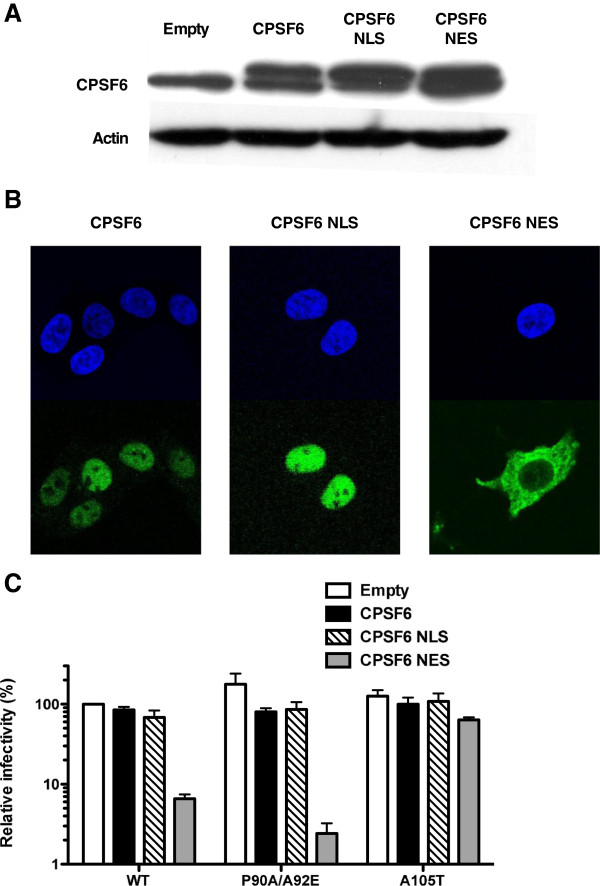
**HIV-1 replication is inhibited when full-length CPSF6 is targeted to the cytoplasm.** (**A**) Expression levels of CPSF6 in TZM-bl cells transduced with empty, CPSF6, CPSF6-NLS and CPSF6-NES vectors. Cell lysates were probed in western blots with anti-CPSF6 antibody (upper panel) and anti-β-actin antibody (lower panel). The upper panel shows the endogenous and exogenous full-length CPSF6 with an HA tag. (**B**) Localization of different forms of CPSF6 in TZM-bl cells stably expressing CPSF6, CPSF6 NLS or CPSF6 NES. The cells were stained with an anti-HA antibody (green) for the detection of the CPSF6 proteins. DAPI staining (blue) was used to mark the nuclear compartment. (**C**) TZM-bl stably expressing the different forms of CPSF6 were challenged with WT or CA mutant HIV-1_NL4-3_GFP reporter viruses. After 72 hours, GFP reporter expression was assessed by flow cytometry. Data represent one of at least three independent experiments. Error bars represent ± SEM (n = 3).

Full-length CPSF6 localizes to the nucleus and when overexpressed does not able to restrict HIV-1 infection. To determine if targeting CPSF6 to the cytosol confers anti-HIV-1 activity, stable cell lines expressing HA-tagged full-length CPSF6 or two modified versions with either an NLS or an NES were produced (Figure 5A). As expected, CPSF6 and CPSF6-NLS localized to the nucleus, and CPSF6-NES was predominantly observed in the cytoplasm (Figure 5B). Infectivity of viruses with WT, A105T and P90A/A92E mutant CA were assessed on these cell lines (Figure 5C). WT virus and the CA mutant P90A/A92E were restricted when CPSF6 was targeted to the cytoplasm (CPSF6-NES). These results demonstrated that, while CPSF6 is a nuclear protein that does not block HIV-1 replication, when it is retargeted to the cytoplasm, HIV-1 replication is inhibited.

### TNPO3 is required for CPSF6 localization to the nucleus and HIV-1 permissiveness

**Figure 6:**
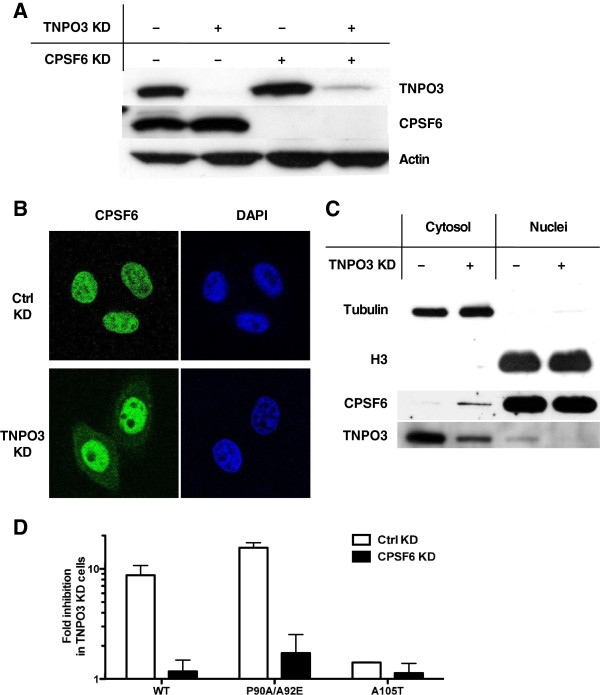
**TNPO3 KD inhibits HIV-1 replication by shifting CPSF6 to the cytoplasm.** (**A**) Expression level of CPSF6 and TNPO3 in TZM-bl cells stably transduced with control or CPSF6 KD vectors and transfected with scrambled or TNPO3 specific small interfering RNA (siRNA). Cell lysate was probed in western blots with anti-TNPO3 antibody (upper panel), anti-CPSF6 antibody (middle panel) and anti-β-actin antibody (lower panel). (**B**) Immuno-fluorescence localization of endogenous CPSF6 (green) in control (Ctrl) KD or TNPO3 KD TZM-bl cells. DAPI staining (blue) was used to mark the nuclear compartment. (**C**) Cell fractionation to identify the cellular localization of endogenous CPSF6. Expression of tubulin in the cytoplasm and histone 3 (H3) in the nucleus was assessed to verify the fractionation. (**D**) Stable ctrl KD and CPSF6 KD cells transfected with scrambled or TNPO3 siRNA were challenged with WT or CA mutant HIV-1_NL4-3_GFP reporter viruses. After 72 hours GFP expression was checked by flow cytometry. The fold inhibition of infectivity due to TNPO3 KD is shown. Data represent one of at least three independent experiments. Error bars represent ± SEM (n = 3).

The RS domain is a NLS for nuclear splicing factors and TNPO3 is a karyopherin that imports SR protein family members [11]. CPSF6-358 lacks the RS domain and, unlike full-length CPSF6, is readily detectable in the cytosol [7]. Together with the finding that HIV-1 is inhibited when full-length CPSF6 is targeted to the cytoplasm, these observations suggested that TNPO3 depletion causes endogenous CPSF6 to accumulate in the cytoplasm, resulting in capsid-specific inhibition of HIV-1, as is seen with CPSF6-358. In a first attempt to test this model, the effect of TNPO3 depletion (Figure 6A) on the subcellular localization of endogenous CPSF6 was examined by immunofluorescence microscopy and cellular fractionation biochemistry. In control knockdown cells, endogenous CPSF6 was detected exclusively in the nucleus (Figure 6B and 6C). When TNPO3 was knocked down, endogenous CPSF6 was also detectable in the cytosol (Figure 6B and 6C).

As a further test of the model, stable CPSF6 knockdown and control knockdown cells were transfected with siRNA targeting TNPO3 or the firefly luciferase gene as a control (Figure 6A), and infectivity of HIV-1 vectors carrying WT CA or CA mutants A105T or P90A/A92E was tested. When CPSF6 was depleted from the cells, TNPO3 KD did not inhibit HIV-1 infectivity (Figure 6D), indicating that CPSF6 was required for the antiviral effect of TNPO3 KD.

**Figure 7:**
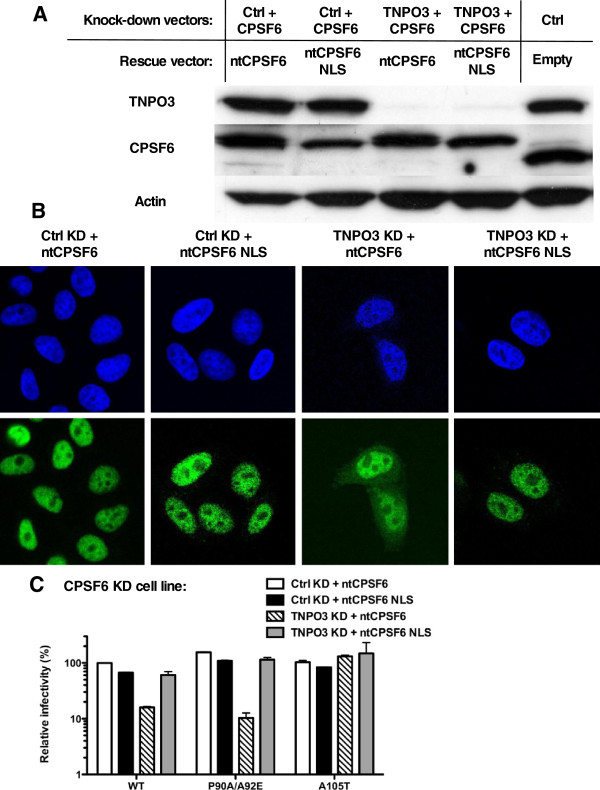
**TNPO3 depletion does not inhibit HIV-1 if CPSF6 is independently targeted to the nucleus.** (**A**) CPSF6 and TNPO3 protein in TZM-bl cells stably transduced with CPSF6 KD vectors, control or TNPO3 KD vectors, and rescue of CPSF6 (ntCPSF6) with or without the SV40 T-Ag NLS. Cell lysate was probed in western blots with anti-TNPO3 antibody (upper panel), anti-CPSF6 antibody (middle panel) and anti-β-actin antibody (lower panel). (**B**) Localization of the non-targetable CPSF6 (ntCPSF6) constructs (green) in control (Ctrl) KD or TNPO3 KD TZM-bl cells stably depleted of CPSF6. DAPI staining (blue) was used to mark the nuclear compartment. (**C**) The pools of stable cell lines in (**B**) were challenged with WT or CA mutant HIV-1_NL4-3_GFP reporter viruses. After 72 hrs, GFP expression was checked by flow cytometry. Data represent one of at least three independent experiments. Error bars represent ± SEM (n = 3).

The CPSF6 stable KD cell line was then transduced with lentiviral vectors encoding either CPSF6 or CPSF6 fused to an NLS, but in each case bearing silent mutations so that they are resistant to the CPSF6 KD vector (ntCPSF6 and ntCPSF6-NLS, respectively). These two stable cell lines were then transduced with a lentiviral vector encoding a modified miRNA that specifically targets either TNPO3, or firefly luciferase as a control (Figure 7A). In control cells, both ntCPSF6 and ntCPSF6-NLS localized to the nucleus (Figure 7B). When TNPO3 was depleted from the cell, ntCPSF6 was found also in the cytoplasm while ntCPSF6-NLS was only detected in the nucleus. This result is consistent with the nuclear localization of CPSF6 being mediated by TNPO3, but when fused to the SV40 T-Ag NLS sequence, CPSF6 uses a different karyopherin in order to localize to the nucleus [34].

The four stable cell lines were then tested for the ability to restrict HIV-1 bearing WT, A105T or P90A/A92E CA (Figure 7C). Conditions where CPSF6 localized to the cytoplasm, resulted in a block on the replication of HIV-1 bearing WT or P90A/A92E mutant CA. These results demonstrate that the decrease in HIV-1 infectivity associated with TNPO3 KD is a consequence of the re-localization of CPSF6 to the cytosol.

### Cytoplasmic CPSF6 stabilizes the HIV-1 CA core

After fusion of HIV-1 with the target cell, the virion core is released into the cytoplasm from which it can be precipitated by ultracentrifugation. CPSF6 binds HIV-1 CA [23] and CPSF6-358 inhibits HIV-1 in a CA-specific manner, with the block occurring at a step before the virus reaches the nucleus (Additional file 1: Figure S1). CPSF6-358 might inhibit HIV-1 infectivity by altering the kinetics of CA core uncoating, consequently delaying the nuclear import of the PIC.

**Figure 8:**
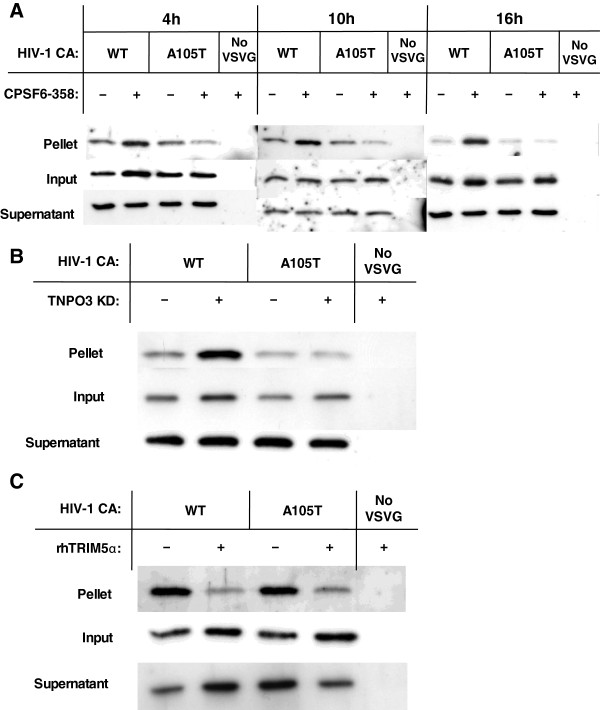
**CPSF6 stabilizes the HIV-1 CA core.** (**A**) Env- HIV-1, pseudotyped with VSV G, and bearing either WT or A105T mutant CA, was incubated with TZM-bl cells stably transduced with CPSF6-358 (+) or empty vector (−), for 4, 10 and 16 hours. As a control, virions lacking VSV G (No VSVG) were also incubated with CPSF6-358 expressing cells. The target cells were lysed and the cytoplasmic fraction (Input) was pelleted through a 50% sucrose cushion to separate the particulate (Pellet) and soluble fractions (Supernatant). The fractions were then analyzed by western blot with an antibody anti-p24. (**B**) WT or A105T CA mutant viruses, and no VSV G virus, were incubated with control (Ctrl) or TNPO3 KD cells for 12 hours. Separation and analysis of the three fractions was described in (**A**). (**C**) WT or A105T CA mutant viruses and Env- virus were incubated with TZM-bl cells stably transduced with rhTRIM5α expressing vector or with an empty vector for 12 hours. Separation and analysis of the three fractions was described in (**A**). All experiments are representative of at least 2 repetitions.

The stability of WT and A105T CA cores in the presence of CPSF6-358 was assessed using a kinetic assay for CA core stability *in vivo* (Figure 8A); variation in the amount of pelletable CA in this assay correlates with altered CA core stability [35]. 4, 10 and 16 hrs after challenge of TZM-bl cells with HIV-1 Env- virus pseudotyped with VSV G, and bearing either WT CA or the A105T CA mutant, cells were lysed and cytoplasmic capsid cores were pelleted through a 50% sucrose cushion. Virus without VSV G was used as a control for CA that had been taken up by cells non-specifically. 4 hrs after challenge with the WT, CA cores showed a slight increase in stability when CPSF6-358 was expressed in the cell; A105T CA core stability was not altered. At 10 and 16 hrs after virus challenge, WT CA core stabilization by CPSF6-358 was even more evident, while the A105T CA core was not altered significantly.

Finally, the effect of TNPO3 KD on the stability of the CA cores was assessed (Figure 8B). WT cores were stabilized when TNPO3 was knocked down, while the CA mutant A105T was not altered. As a positive control, destabilization of the CA core mediated by rhTRIM5α was assessed [35]. Both WT and A105T CA cores were destabilized when rhTRIM5α was expressed (Figure 8C). These results indicate that retention of CPSF6 in the cytoplasm, either via deletion of its NLS or KD of TNPO3, inhibits HIV-1 replication by causing hyperstabilization of the CA core, and presumably delaying transit of the PIC to the nucleus.

## Discussion

### TNPO3 KD inhibits HIV-1 in a step before nuclear import

In previous works, when the effect of TNPO3 on HIV-1 replication was assessed, some research groups showed that TNPO3 promotes HIV-1 replication at a step before nuclear import, while an equal number claimed that it acts after nuclear entry [5,6,8,9,12-17]. The assay for HIV-1 nuclear import that was used by all of these investigators was PCR-based detection of 2-LTR circles [26]. These circular viral cDNAs are generated by cellular enzymes that promote the covalent joining of the LTR termini [36]. In the work here, the PCR products amplified using standard primers flanking the 2-LTR circle junction were examined in detail. As previously described, 2-LTR circles with consensus sequence, deletion of the termini, point mutations and PBS or PPT insertions were observed [27,28]. We also identified abundant products (between 45 and 58% of the total) amplified from circles produced during opposite-strand joining autointegration events. When we sequenced the unintegrated viral products present in the cell after HIV-1 infection, we saw between 600 and 5000 times more autointegrants compared to 2-LTR circles. Differences in the assessment of 2-LTRs could easily be explained by interference from the abundant autointegration products.

When 2-LTR PCR products were cloned and sequenced, the bona fide 2-LTR circles with consensus LTR junctions were significantly reduced when TNPO3 was depleted. We then designed new primers that are able to discriminate consensus 2-LTR circles from autointegration events. Using this PCR assay and the deep-sequencing analysis of unintegrated viral products, we again demonstrated a significant reduction of 2-LTR circles as a result of TNPO3 depletion. Lack of specificity of the standard 2‐LTR circle PCR therefore explains why different research groups have not obtained similar data. Indeed, all studies that reported no difference in the level of 2‐LTR circles by TNPO3 depletion used primers flanking the circle junction to detect 2‐LTR circles [8,12,13,15,16]. Many studies that have quantified the 2-LTR circles using primers that flank the circle junction should be revisited with the new PCR assay.

### TNPO3 prevents CPSF6 from inhibiting HIV-1 replication

A model that describes the mechanism of action of TNPO3 in HIV-1 replication has remained elusive for several years. Based on their observation that TNPO3 interacts with HIV-1 IN, Christ et al. hypothesized that TNPO3 has a direct role in nuclear import of the viral preintegration complex [6]. Then several groups demonstrated that HIV-1 capsid is the primary determinant of TNPO3-dependence [8,19]. However, more recent reports suggest that TNPO3 might affect HIV-1 nuclear import only indirectly [9,14]. Zhou et al. proposed that TNPO3 promotes HIV-1 replication after the PIC has been imported into the nucleus, by displacing and exporting to the cytosol the viral CA still associated with the nuclear PIC [13]. In our previous work, analyzing a panel of CA mutants, we hypothesized that TNPO3 might alter the stability of the CA core [8].

Interestingly, Lee et al. recently characterized a truncated form of CPSF6, CPSF6-358, capable of restricting HIV-1 infectivity in a CA-dependent manner similarly to TNPO3 [7]. CPSF6-358 lacks the C-terminal RS-like domain, a NLS region commonly used by cargo proteins for a TNPO3-dependent nuclear import. Lee et al. showed that full-length CPSF6 is strictly localized to the nucleus of NIH3T3 cells, while the truncated protein is also present in the cytosol, suggesting that the RS-domain is required for the nuclear localization of the protein [7]. Here we showed that CPSF6 is indeed capable of inhibiting HIV-1 replication, but only when it accumulates in the cytosol, as occurs when TNPO3 is disrupted. Our data suggest that when CPSF6 accumulates in the cytosol, it binds to CA via the pocket where N74 and A105 are located [7]. This stabilizes the HIV-1 CA core, causing a delay in uncoating and in nuclear import of the viral cDNA.

Since results obtained in our study are based on the artificial alteration of CPSF6 localization, the question now remains: is there a physiological condition where CPSF6 accumulates in the cytoplasm to control HIV-1 replication? A recent study reported that, in response to Toll-like receptor-3 (TLR-3) stimulation, macrophages counteract HIV-1 by upregulating microRNA-155; this has the result that several factors important to the early steps of HIV-1 replication - including TNPO3 - are down-regulated [37]. Further studies are required to find an answer to this question. However, we can hypothesize two scenarios: CPSF6 could become cytoplasmic and restrict HIV-1 infection in specific cell types or after particular stimuli; CPSF6 is a factor required for proper CA core uncoating in certain conditions.

### CA core stability is tightly regulated by host cellular factors

Since the discovery of the interaction between HIV-1 CA and the host peptidyl-prolyl isomerase cyclophilin A (CypA) [38], several host factors that bind HIV-1 CA and alter the early steps of virus replication have been identified [7,9,33,39]. The nuclear pore protein Nup358 seems to be directly involved in promoting the nuclear import of the HIV-1 PIC, while CypA and the tripartite motif 5 (TRIM5) proteins have been demonstrated to regulate the stability of the CA cores. The TRIM5 proteins specifically recognize the CA core lattice and accelerate uncoating, causing a premature release of the reverse transcription complex (RTC) and consequently inhibits viral cDNA synthesis [35,40]. CypA has opposite effects on HIV-1 replication in different cell lines: in Jurkat T cells CypA stabilizes CA cores promoting reverse transcription, in HeLa cells CypA destabilizes A92E CA mutant cores blocking viral replication in a step between reverse transcription and integration [41-43]. The impact of these cellular factors on HIV-1 replication, together with the inhibition of reverse transcription by CA mutants that alter core stability [44], demonstrates how crucial is the optimal stability of the CA cores for productive infection. Recent work also demonstrated a direct correlation between HIV-1 reverse transcription and core uncoating [3]. In this study, we characterized a new host factor that alters CA core stability, CPSF6. The SR-protein specifically binds a pocket located in the N-terminal domain of HIV-1 CA previously described as the target of a compound capable of destabilizing CA cores, and stabilizes the cores [23,45].

## Conclusions

Since the identification of TNPO3 as a factor required for HIV-1 infection many studies have attempted to understand the specific function of this karyopherin in viral replication. Experiments in this manuscript demonstrate that TNPO3 is not directly required for nuclear import of the viral PIC. Instead, TNPO3 promotes HIV-1 infection indirectly, by inhibiting an inhibitor of HIV-1, CSPF6. In the presence of TNPO3, CPSF6 localizes to the nucleus. When TNPO3 is depleted, CPSF6 accumulates in the cytoplasm where it stabilizes HIV-1 CA cores and prevents their disassembly.

## Methods

### Cell lines, tissue culture, and drugs

293T and TZM-bl cells were grown in Dulbecco’s modified Eagle medium (DMEM) (Invitrogen) supplemented with 10% fetal bovine serum (FBS) (PAA), 20 mM L-glutamine, 1000 U/ml penicillin, and 1000 mg/ml Streptomycin (GIBCO). Raltegravir (RAL, Santa Cruz) was used at a final concentration of 10 μM.

### Plasmids

pWPTs-GFP is an HIV-1-based transfer vector with EGFP expression under the control of the EF1α promoter [46]. p8.9NdSB is a minimal HIV-1 packaging plasmid for *gag* and *pol* expression [47]. pMD2-G encodes the vesicular stomatitis virus G protein (VSV-G). pNL4-3.GFP.E^−^ bears HIV-1 proviral sequence with an *env*-inactivating mutation and EGFP in place of *nef*[48]. pAPM and pAHM are HIV-1 based knockdown vector in which a single transcript driven by the spleen focus-forming virus (SFFV) LTR contains a miR30 framework modified to target a gene of interest and the puromycin N-acetyltransferase gene or hygromycin B phosphotransferase, respectively [49]. pAIB is an HIV-1-based transfer vector expressing the protein of interest from the SFFV LTR followed by the encephalomyocarditis virus (EMCV) internal ribosome entry site (IRES) cassette and the drug resistance cassette, blasticidin-S-deaminase [50]. CPSF6 and CPSF6-358 were subcloned from pLPC-CPSF6-FL-HA and pLPC-CPSF6-358-HA (kindly provided by Vineet KewalRamani) into pAIB by cutting pAIB and pLPC with NotI/XbaI and BglII/NotI respectively [7]. pCR®II-TOPO® (Invitrogen) is a plasmid containing single 3′-thymidine overhangs for TA cloning and the topoisomerase I enzyme covalently bound to the vector.

### Knockdown vector cloning and siRNA transfection

AHM-CPSF6 vectors targeting three different sequences of CPSF6 were designed as previously described [8]. Three 97-mer oligonucleotides were synthesized and PAGE purified: ts10, 5′-TGCTGTTGACAGTGAGCGCGGAAAGA GAATTGCATTATATTAGTGAAGCCACAGATGTAAT ATAATGCAATTCTCTTTCCATGCCTACTGCCTCGG A-3′; ts13, 5′- TGCTGTTGACAGTGAGCGAGCAATC TCAAGCAGTGCTATTTAGTGAAGCCACAGATGTAA ATAGCACTGCTTGAGATTGCCTGCCTACTGCCTCG GA-3′; ts37, 5′-TGCTGTTGACAGTGAGCGATTGGA TCTGAAGCATCTTCAATAGTGAAGCCACAGATGTA TTGAAGATGCTTCAGATCCAACTGCCTACTGCCTC GGA-3′. TZM-bl cells were transduced with the AHM-CPSF6 vectors (ts10, ts13 and ts37). Efficiency of the CPSF6 KDs was analyzed by western blot and AHM-CPSF6 ts10 was selected since it was the most potent of the three constructs. The APM-TNPO3 KD vector and the TNPO3 siRNA transfection procedure were previously described [8].

### Cloning and mutagenesis

To express a form of CPSF6 that was resistant to CPSF6 KD ts 10, silent mutations were introduced into the CPSF6 cDNA (ntCPSF6) by overlapping PCR using the oligonucleotides ntCPSF6 fwd, 5′- CAAATGTTGTCTA TACATATACTGGAAAACGTATAGCGCTTTATATTG GAAATCTAACATGGTGGAC -3′; ntCPSF6 rev, 5′- G TCCACCATGTTAGATTTCCAATATAAAGCGCTATA CGTTTTCCAGTATATGTATAGACAACATTTG -3′.

NLS and NES were cloned at the C-terminus of CPSF6, ntCPSF6 and CPSF6-358. PCR used pAIB-CPSF6-HA, pAIB-ntCPSF6-HA and pAIB-CPSF6-358-HA as templates and the primers SSFV (fwd), 5′-C TCACTCGGCGCGCCAGTC -3′ coupled with SV40 NLS (rev), 5′- TGTGTGGCGGCCGCCTATACCTTTC TCTTCTTTTTTGGGGCGTAGTCGGGCACGTC -3′; or PKI NES (rev), 5′- TGTGTGGCGGCCGCCTAATT TATATCGAGTCCAGCTAGCTTCAATGCCAGGGCG TAGTCGGGCACGTC -3′. The PCR products were subcloned into pAIB-CPSF6-HA, pAIB-ntCPSF6-HA and pAIB-CPSF6-358-HA vectors digesting with NotI and XbaI.

### Tranduction and transfection of mammalian cells

To express the different forms of CPSF6, TZM-bl cells were transduced with pAIB encoding CPSF6-358, CPSF6-358-NLS, CPSF6-358-NES, CPSF6, CPSF6-NLS or CPSF6-NES. To express rhTRIM5 or hTRIMCyp, cells were transduced with pAIB encoding the respective proteins. Cells were selected with blasticidin (10 μg/mL) starting two days after transduction.

To generate stable KDs, TZM-bl cells were transduced with pAHM microRNA-based shRNA vectors targeting either control or CPSF6 mRNA. Cells were selected with 200 μg/mL of hygromycin B two days after transduction. To generate the rescue cells, CPSF6 KD cells were transduced with the pAIB vector encoding for ntCPSF6 or ntCPSF6-NLS. 2 days after transduction, the cells were selected with 10 μg/mL blasticidin.

For transfection of siRNA, Lipofectamine RNAiMAX was complexed with 100 nM of Gene Solution siRNA (Qiagen) targeting TNPO3 following manufacturer’s instruction.

### Production of viruses and vectors

Viruses and minimal vectors were produced by transfection of 293T cells using Polyethylenimine (PEI) (Sigma, Inc) as previously described [8].

### Western blot analysis

We used rabbit anti-TNPO3 antibody (ab71388, abcam), rabbit anti-CPSF6 antibody (Novus biological), human anti-p24 (NIBSC) and mouse anti-actin antibody (Sigma). The secondary antibodies were HRP-linked donkey anti-human IgG (Jackson), HRP-linked donkey anti-rabbit IgG and HRP-linked sheep anti-mouse IgG (GE Healthcare Life Sciences).

### 2-LTR circles, late RT and autointegration PCR

Low molecular weight DNA was extracted from 4 × 10^6^ cells using the QIAprep Spin Miniprep Kit (Qiagen), following the manufacturer’s instructions.

Quantitative PCR for NL4.3 GFP E- 2-LTR circles was detected with Sybr green (Invitrogen) or TaqMan probes. 2-LTR circle detection with primers external to the circle junction (Figure 1B) was made with primers: NL4.3_2 fwd, 5′-GAGATCCCTCAGACCCTTTTAG-3′; MH536, 5′- TCCACAGATCAAGGATATCTTGT C-3′. The primers used for detection of 2-LTR circles with perfect junction (Figure 1F) are: Junct2 fwd, 5′-C AGTGTGGAAAATCTCTAGCAGTAC-3′; or Junct4 fwd, 5′- CAGTGTGGAAAATCTCTAGCAGTACTG-3′; both coupled with J2 rev, 5′-GCCGTGCGCGCTT CAGCAAGC-3′. 2-LTR circles qPCRs with TaqMan probe detection method were made using the primers [26]: MH535, 5′-AACTAGGGAACCCACTGCTTAA G-3′; and MH536. The TaqMan probes external or overlapping with the junction were, respectively: MH603, 5′-(FAM)-ACACTACTTGAAGCACTCAAGG CAAGCTTT-(TAMRA)-3′ [26]; JunctPro, 5′- (FAM)-C TCTAGCAGTACTGGAAGGGCTA-(TAMRA)-3′.

Sybr green 2-LTR circle PCR reaction contained 1x Sybr green mix (10 mM Tris pH 8.3, 10 mM KCl, 2.5mM NH_4_SO_4_, 5 mM MgCl_2_, 0.1 mg/ml BSA, 0.2 mM dNTPs, 1× Sybr green), 300 nM each primer, 6 μl of template low-molecular weight DNA, and 0.2 μl of Hot Start Taq Polymerase (Promega) in a volume of 20 μl. After initial incubation at 95°C for 2 min to activate the Hot Start Taq Polymerase, 40 cycles of amplification and acquisition were carried out at 95°C for 6 s, followed by 10 s at 55°C, 30 s at 72°C and 6 s at 80°C. TaqMan 2-LTR circle PCR reaction mix contained 1× TaqMan Universal Master Mix (Applied Biosystems), 50 nM primer forward and reverse, 100 nM TaqMan probe and 6 μl of template low-molecular weight DNA in a volume of 20 μl. After an initial incubation at 95°C for 10 min, 40 cycles of amplification were carried out at 95°C for 15 s followed by 1 min and 30 s at 60°C. qPCR reactions were made using the CFX96™ thermal cycler (Biorad). Where indicated, cells were treated 1 hour before infection with 10 μM Raltegravir.

Late RT levels were assessed by qPCR as previously described [8].

Autointegration events were measured by two-step nested PCR as described [31]. The first amplification step used 200 ng of genomic DNA, 1X PCR buffer, 1.5 mM MgCl_2_, 0.2 mM of dNTPs, 0.5 μl Hot Start Taq polymerase, 0.2 μM of primers PBS- (5′ - TTTCCGGTCCCTGTT CGGGCGCCA- 3′), NY199/primer B- (5′ – CTACCTTG TTATGTCCTGCTTG- 3′) and NY200/primer A + (5′- CTCTACAGCACTTGGCACTAGC- 3′) in a final volume of 25 μl. After initial incubation at 95°C for 5 min, 24 cycles of amplification were carried out at 95°C for 30 s followed by 30 s at 60°C and 3 min at 72°C; a final step of elongation was made at 72°C for 7 min. The PCR product was then diluted 1:100 and used in the second step of amplification carried out with the same conditions and primers (NL4.3_2 fwd and MH536) as the qPCR for the detection of 2-LTR circles using primers external to the circle junction.

### Cloning and sequencing of PCR products

2-LTR circle PCR products were cloned into pCR®II-TOPO® by TOPO TA cloning (Invitrogen), following the manufacturer’s instructions. The plasmids were transformed into One Shot Mach1-T1 competent cells and plated in petri dishes. Single colonies were picked and used to stab a 96 well agar plate. Sequencing was carried out by Beckman Genomics (UK).

### High-throughput sequencing of viral DNA byproducts

Genomic libraries were prepared using the TruSeq® DNA Sample Prep kit V2 (Illumina) following manufacturer’s instructions. Briefly, 1 μg of genomic DNA was sheared with the Covaris 2 system (Covaris). The DNA fragments were then end-repaired, extended with an ‘A’ base on the 3′ end, ligated with indexed paired-end adaptors and PCR amplified. PCR amplification was carried out as follows: initial denaturation at 98°C for 30 sec, followed by 8 cycles consisting of 98°C for 10 sec, 60°C for 30 sec and 72°C for 30 sec, then a final elongation at 72°C for 5 min. Four different genomic libraries were pooled and sequenced in one lane of an Illumina HiSeq2000 sequencer using a 2 x 95bp paired end indexing protocol. Demultiplexed fastq files were obtained for each sample using the Illumina CASAVAv1.8.1 software and processed by a custom pipeline running on the Vital-IT (http://www.vital-it.ch). Center for high-performance computing of the SIB Swiss Institute of Bioinformatics. Specifically, the mapping of fastq reads has been performed with BWA [51] with duplicate removal by samtools [52] against the HIV genome in order to quantify the amount of virus for each sample. The same fastq reads have then been mapped against the human genome to eliminate DNA cell contamination (>90%). Remaining reads have been finally processed with Blat [53] and custom made Python scripts against HIV genome to capture and count the occurrence of junction structures as circles and autointegrants.

Average coverage of the HIV genome is ~5000 and the number of detected junctions (90% autointegrants) ranges from 30000 to 100000 according to the viral quantity (Table S1).

The complete high throughput sequencing dataset has been submitted to the Sequence Read Archive (SRA) /ci(http://www.ncbi.nlm.nih.gov/sra) under accession number SRA056122.

### Immunofluorescence assay

TZM-bl cells expressing specific KD vectors or a version of the HA-tagged CPSF6 protein were fixed for 10 min with 3% paraformaldhyde, quenched for 10 min with 0.1 M glycine, permeabilized for 10 min with 0.2% Triton-X 100, and blocked for 20 min with 1% BSA in PBS. Cells were then incubated for 1 hour either with anti-HA (Covance) or anti-CPSF6 antibodies (Novus Biologicals) diluted in PBS with 1% BSA. After 5 washes, the cells were incubated with anti-mouse (HA) or anti-rabbit (CPSF6) Alexa Fluor 488-conjugated secondary antibodies for 1 hour, washed again, incubated for 1 min in Hoechst and mounted with Mowiol. The slides were viewed with a Zeiss LSM 510 confocal laser-scanning microscope.

### Cellular fractionation

Control and TNPO3 KD TZM-bl cells were resuspended in 1 ml of ice-cold hypotonic buffer (10 mM HEPES pH 7.9, 1.5 mM MgCl_2_, 10 mM KCl, 0.5 mM DTT, protease inhibitors) and lysed in a 7-ml Dounce homogenizer by 15 stokes with pestle B. The lysate was centrifuged at 1,000 rpm for 5 min at 4°C and the supernatant was used as cytoplasmic fraction. The nuclear pellet was resuspended in 1 ml of sucrose buffer (0.25 mM sucrose, 10 mM MgCl_2_, protease inhibitors), layered over a 1 ml sucrose cushion (0.88 mM sucrose, 0.5 mM MgCl_2_, protease inhibitors) and centrifuged at 3,500 rpm for 10 min at 4°C. The nuclear pellet was finally resuspended in 1× SDS-PAGE loading buffer.

### Fate of capsid assay

Fate of capsid assay was performed as previously described [35]. TZM-bl cells expressing an empty vector, CPSF6-358, rhTRIM5α or the TNPO3 KD were seeded onto T75 flasks. 24 hours later, the confluent cells were incubated with 10 ml of Env- HIV-1, pseudotyped with VSV G, and bearing either WT or A105T mutant CA, for 30 min at 4°C and then shifted to 37°C. After 4 hours, the virus was removed, the cells were washed and returned to 37°C for 12 hours. Cells were detached with pronase (7 mg/ml in DMEM) for 5 min at 4°C, washed 3 times with ice cold PBS and finally resuspended in 2.5 ml of hypotonic lysis buffer (10 mM Tris–HCl, pH 8.0, 10 mM KCl, 1 mM EDTA). After 5 min incubation on ice, the cells were lysed in a 7-ml Dounce homogenizer by 15 stokes with pestle B. The lysate was cleared by centrifugation for 3 min at 3,000 rpm at 4°C to remove the nuclear fraction. 100 μl of the cleared lysate was collected to determine the viral input in the assay, while 2 ml was layered on top of a 7-ml 50% sucrose gradient and centrifuged for 2 hours at 30,000 rpm at 4°C using a Beckman SW41 rotor. After centrifugation, 100 μl of the top-most part were collected (supernatant) and the pellet resuspended in 1× SDS-PAGE loading buffer. All samples were analyzed by WB using antibody against p24.

### Ethics statement

All experimental protocols were approved by the ethical committees of the University of Geneva and the University of Massachusetts.

## Competing interests

The authors declare that they have no competing interests.

## Authors’ contributions

AD and JL conceived and designed the experiments and wrote the paper. AD, AV, and MG performed the experiments. AD, FS, SA, and JL analyzed the data. All authors read and approved the final manuscript.

## Supplementary Material

Additional file 1 **Supplemental data. Table S1** Represents the raw data of Figure 1D. **Figure S1** Extension of the characterization of 2-LTR circles formed in absence of TNPO3 and characterization of 2-LTR circles formed in presence of CPSF6-358. **Figure S2** Lack of correlation of inhibition of the 27 HIV-1 CA mutants analyzed in Figure 2C infecting CPSF6-358 expressing cells or cells expressing other restriction factor. **Table S2** represents some details of the deep sequencing analysis from Figure 1J. Click here for file
