# Supplementary material for: TNPO3 protects HIV-1 replication from CPSF6-mediated capsid stabilization in the host cell cytoplasm
Source: Retrovirology. 2013 Feb 15;10:20. doi: 10.1186/1742-4690-10-20 (PMC3599327; doi:10.1186/1742-4690-10-20)
Supplement: Additional file 1 — Supplemental data. Table S1 Represents the raw data of Figure 1D. Figure S1 Extension of the characterization of 2-LTR circles formed in absence of TNPO3 and characterization of 2-LTR circles formed in presence of CPSF6-358. Figure S2 Lack of correlation of inhibition of the 27 HIV-1 CA mutants analyzed in Figure 2C infecting CPSF6-358 expressing cells or cells expressing other restriction factor. Table S2 represents some details of the deep sequencing analysis from Figure 1J. [file 1742-4690-10-20-S1.docx]

**Supplemental Information**

**TNPO3 protects HIV-1 replication from CPSF6-mediated capsid stabilization in the host cell cytoplasm**

Alberto De Iaco, Federico Santoni, Michel Guipponi, Stylianos Antonarakis and Jeremy Luban

1. **Supplemental Data**

Table S1 Represents the raw data of Figure 1D

Figure S1 Extension of the characterization of 2-LTR circles formed in absence of TNPO3 and characterization of 2-LTR circles formed in presence of CPSF6-358

Figure S2 Lack of correlation of inhibition of the 27 HIV-1 CA mutants analyzed in figure 2C infecting CPSF6-358 expressing cells or cells expressing other restriction factor

Table S2 Represents some details of the deep sequencing analysis from Figure 1J

1. **Supplemental References**
2. **Supplemental Data**

***Table S1, Related to Figure 1***


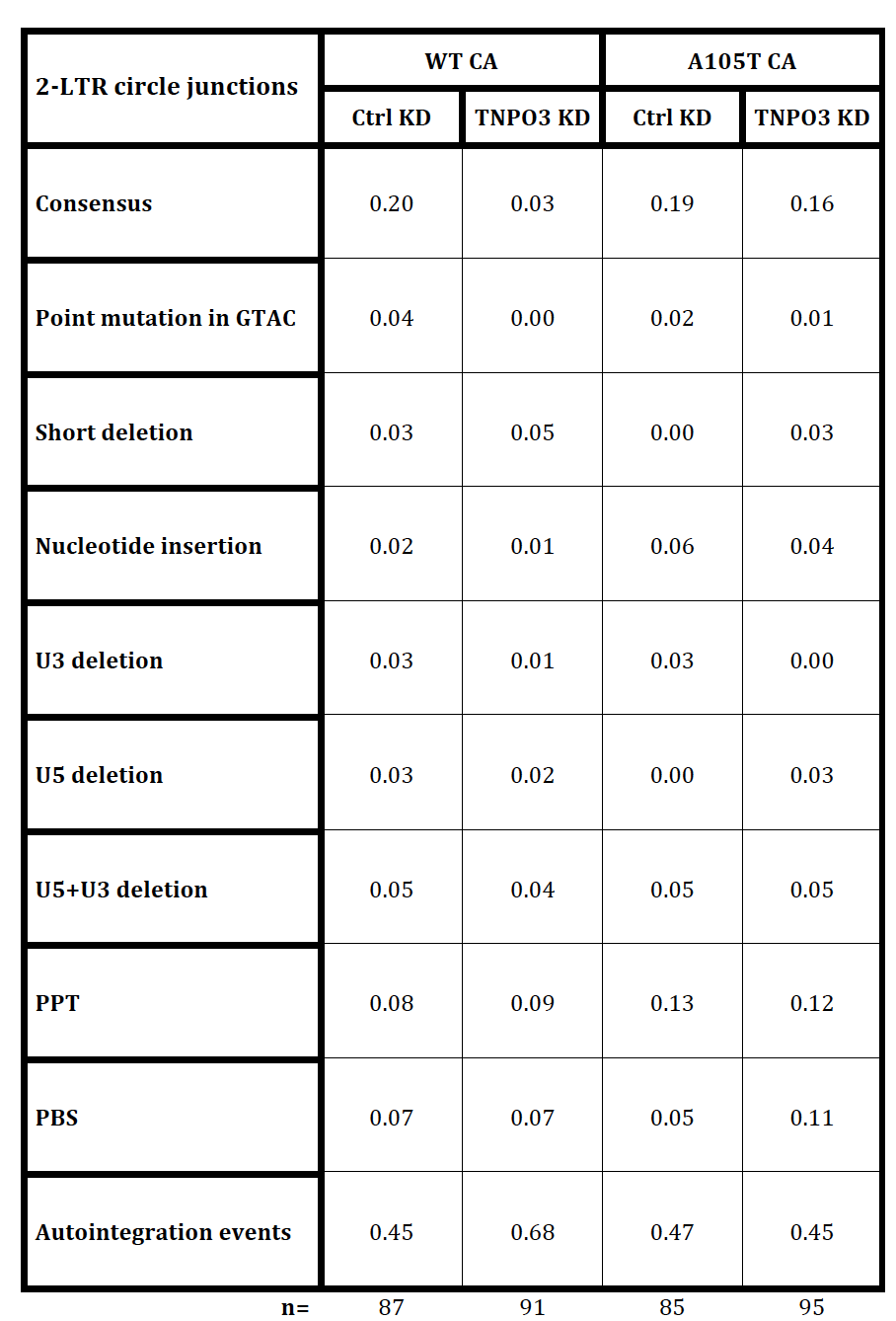


**Sanger sequencing of the 2-LTR circle products from PCR using primers flanking the circle junction**

2-LTR circle PCR products from Figure 1C were cloned into bacterial plasmids and single colonies were sequenced using a primer overlapping with the T7 promoter. The numbers in the boxes here are the relative frequencies of sequences identified for each type of junction, where 100% equals 1.0. The total number of plasmids sequenced for each condition are represented by n.

***Figure S1, Related to Figure 1***

***
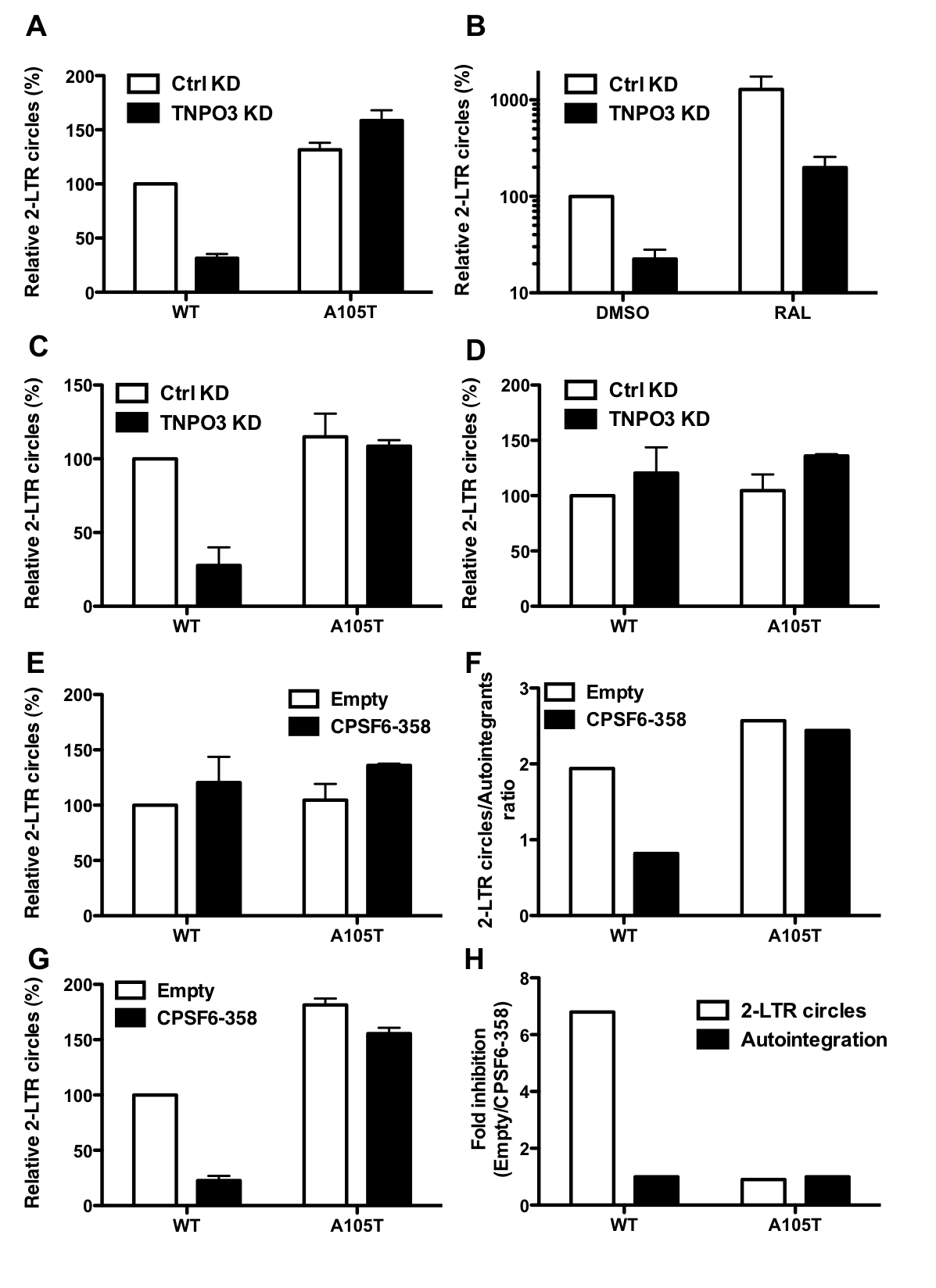
***

**Inhibition of 2-LTR circle formation in the absence of TNPO3 or in the presence of CPSF6-358, assessed with different qPCR methods**

**(A)** Quantification of 2-LTR circle PCR products with forward primer overlapping with 4 nucleotides of the 5’LTR (as indicated in Figure 1G, junct4 fwd) after 24 hours infection of WT and A105T CA mutant viruses on control (Ctrl) or TNPO3 KD TZM-bl cells. **(B)** Quantification of 2-LTR circle PCR products with forward primer overlapping the junction (junct2 fwd) after 24 hours infection of WT virus on control (Ctrl) or TNPO3 KD TZM-bl cells, treated with raltegravir (RAL) or DMSO as a control. **(C)** Quantification of 2-LTR circles using primers flanking the circle junction and a TaqMan probe annealing with the 3’LTR [[1](#_ENREF_1)] **(D)** Quantification of 2-LTR circles using primers flanking the circle junction and a TaqMan probe annealing within the junction sequence. **(E)** Quantification of 2-LTR circle PCR products (from analysis represented in figure 1B) after 24 hours infection of WT and A105T CA mutant viruses on TZM-bl cells expressing or not CPSF6-358. **(F)** The PCR products were cloned and sequenced, and the amount of 2-LTR circles and autointegration events determined. The ratios between 2-LTR circles and autointegration events detected were plotted. **(G)** Quantification of 2-LTR circle PCR products with forward primer overlapping with 2 nucleotides of the 5’LTR (as indicated in figure 1G) after 24 hours infection of WT and A105T CA mutant viruses on control (Ctrl) or TNPO3 KD TZM-bl cells. **(H)** 2-LTR circles and autointegration events are quantified by high-throughput sequencing of low-molecular weight DNA extracted from TZM-bl stably transduced with an vector expressing CPSF6-358 or an empty vector as a control infected for 24 hours with WT or A105T CA mutant viruses. Ratios between the levels in empty vs CPSF6-358 expressing cells are plotted. Data represent one of at least three independent experiments. Error bars represent ± SEM (n = 3).

***Figure S2, Related to Figure 2***


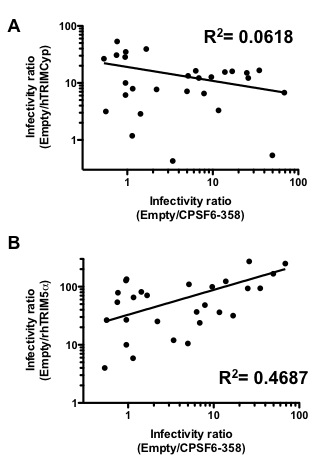


**CPSF6-358 inhibition of HIV-1 CA mutants does not correlate with the restriction of other CA-dependent restriction factors**

**(A)** TZM-bl cells stably transduced with human TRIMCyp (hTRIMCyp) expressing vector or empty vector were challenged with 27 CA mutant HIV-1 vectors. Correlation between the infectivity ratios of 27 CA mutants infecting Empty vs hTRIMCyp and Empty vs CPSF6-358 (Figure 2C) (R^2^=0.0618). **(B)** TZM-bl cells stably transduced with rhesus TRIM5α (rhTRIM5α) expressing vector or empty vector were challenged with 27 CA mutant HIV-1 vectors. Correlation between the infectivity ratios of 27 CA mutants infecting Empty vs rhTRIM5α and Empty vs CPSF6-358 (Figure 2C) (R^2^=0.4687). Data represent one of at least three independent experiments. Error bars represent ± SEM (n = 3).

***Table S2, Related to Material and Methods***

|  | **# mapped reads** | **Coverage** | **2-LTR Circles** | **2-LTR Circles**  **WT Ctrl normalized^*^** | **2-LTR Circles**  **Norm Fold Change*** |
| --- | --- | --- | --- | --- | --- |
| **WT**  **Ctrl^*^** | 1502117 | 14401 | 217 | 217 | 1 |
| **A105T**  **Ctrl** | 745319 | 7146 | 113 | 227 | 0.96 |
| **WT**  **TNPO3 KD** | 2240139 | 21477 | 91 | 61 | 3.56 |
| **A105T**  **TNPO3 KD** | 945335 | 9063 | 167 | 265 | 0.82 |

|  | **# mapped reads** | **Coverage** | **2-LTR Circles** | **2-LTR Circles**  **WT Ctrl normalized^**^** | **2-LTR Circles**  **Norm Fold Change**** |
| --- | --- | --- | --- | --- | --- |
| **WT**  **Ctrl^**^** | 465114 | 4459 | 55 | 55 | 1 |
| **A105T**  **Ctrl** | 946378 | 9074 | 134 | 65 | 0.84 |
| **WT**  **CPSF6-358** | 1056108 | 10126 | 18 | 8 | 6.8 |
| **A105T**  **CPSF6-358** | 487368 | 4672 | 56 | 53 | 0.96 |

**Mapping statistics, coverage and number of 2-LTR circles of whole-genome and junctions high throughput sequencing of WT and A105T mutant preintegration complex on control or TNPO3 KD or CPSF6-358 overexpressing TZM-bl cells.** Coverage (number of reads X nucleotide) is calculated by dividing the number of mapping reads by the length in nt. of HIV-1_NL4-3_ multiplied by the read length (100nt). 2-LTR Circles are quantified by counting reads covering 3’LTR-5’LTR junction and containing CTGACTG sequence (or its reverse complement if they cover 5’LTR-3’LTR junction). 2-LTR circles are normalized to corresponding WT Ctrl and Fold Change wrt WT Ctrl is calculated accordingly.

1. **Supplemental References**

1. Butler SL, Hansen MS, Bushman FD: **A quantitative assay for HIV DNA integration in vivo.** *Nat Med* 2001, **7:**631-634.
